# The Prognostic Value of Reticulated Platelets in Patients With Coronary Artery Disease: A Systematic Review and Meta-Analysis

**DOI:** 10.3389/fcvm.2020.578041

**Published:** 2020-10-23

**Authors:** Yihan Zhao, Runmin Lai, Ying Zhang, Dazhuo Shi

**Affiliations:** ^1^Beijing University of Chinese Medicine, Beijing, China; ^2^Cardiovascular Diseases Center, Xiyuan Hospital, China Academy of Chinese Medical Sciences, Beijing, China

**Keywords:** reticulated platelets, coronary artery disease, cardiovascular events, prognostic value, meta-analysis

## Abstract

**Background:** Reticulated platelets (RPs) represent the young population in the circulating platelet pool, indicating platelet turnover. Preliminary studies suggested circulating levels of RPs were associated with cardiovascular events (CVEs) in patients with coronary artery disease (CAD).

**Methods:** This study systematically searched PubMed, Scopus, Embase, and Web of Science for eligible studies which reported RPs as a prognostic factor and the incidence of CVEs in patients with CAD. The risk estimates and 95% confidence intervals (95% CI) were analyzed for adjusted and unadjusted associations separately using random-effects model. Meta-regression and subgroup analysis were used to identify the source of heterogeneity. Funnel plots, Egger's test, and trim and fill methods were used to assess the publication bias.

**Results:** A total of six cohort studies were included in this meta-analysis. Four studies were rated as high quality with the remaining rated as moderate quality. The funnel plot, Egger's test, and trim and fill method suggested the presence of publication bias. The pooled results indicated elevated RPs were associated with a higher risk of composite CVEs [risk ratio (RR), 2.26; 95% CI, 1.72–2.98, with little heterogeneity] and cardiovascular death (RR, 2.33; 95% CI, 1.66–3.28, with little heterogeneity). Based on results of separate meta-analysis, we found RPs might be a good predictor for revascularization but not for myocardial infarction or cerebrovascular events. After adjustment of conventional prognostic factors, the pooled result still suggested the prognostic value of RPs for composite CVEs (RR, 2.00; 95% CI, 1.30–3.08; *p* < 0.00001, with substantial heterogeneity). Subgroup analysis and meta-regression of adjusted risk estimates revealed that the number of adjustment factors might be the source heterogeneity.

**Conclusion:** Circulating level of RPs might be a useful prognostic marker for CVEs in patients with CAD, even after adjustment of other prognostic factors.

## Introduction

Patients with coronary artery disease (CAD) face considerable risk of cardiovascular events (CVEs) despite the wide application of antiplatelet therapy (1). As included in commonly used prediction scores, many conventional factors are used to predict the prognosis of patients with CAD (2). In addition to conventional factors, there is a growing body of evidence demonstrating that high on-treatment platelet reactivity due to insufficient response to antiplatelet therapy is critically linked to adverse clinical events (3–5). One of the plausible mechanisms contributing to high on-treatment platelet reactivity includes the enhanced platelet turnover. A broad array of platelet turnover tests is available in clinical practice, like mean platelet volume (MPV) and platelet function tests. In addition to these laboratory parameters widely available for decades, a novel parameter, reticulated platelets (RPs) could also act as a surrogate of platelet turnover (6).

The platelet population is not homogeneous, and RPs comprise the youngest population in the circulating platelet pool. Unlike mature platelets, nucleic acid-rich RPs have larger sizes, contain more dense granules, and have increased thrombotic activity (7–9). These newly released platelets also have intrinsic and functional properties, which lead to over-proportionate and persisted aggregation formation (10). RPs act as seeds for the formation of aggregates and locate the core of aggregates (10). Initially, RPs were identified with flow cytometry after the thiazole orange staining (11). However, the time-consuming flow cytometry measurement without a standardized protocol restrained its broader application in clinical practice (12). Recently, a newer assay (SysmexXE-2100 or 5000 hematology analyzer) has become an alternative to flow cytometry. The fluorescent dye used in this technique penetrates cell membranes and stains platelet RNA. The stained platelets are then sorted through a semiconductor laser diode. With the results of cell volume (measured by forward scattered light) and RNA content (measured by fluorescence intensity), the system could separate the mature platelets from immature platelets. RPs separated by this method are expressed as immature platelet fraction (IPF%) or immature platelet count (IPC). IPC represents the absolute number of RPs and can be calculated by multiplying the IPF by the platelet count (6). This novel automated analyzer of immature platelet has been developed for the quantification of immature platelet determination as part of the complete blood count with little added cost (13, 14).

However, unlike MPV, the conventional parameter of platelet turnover, which has been extensively studied (15, 16), little is known about the prognostic value and optimal cut-off point of RPs specifically in CAD. Therefore, we carried out this systematic review and meta-analysis to determine the following: (1) whether association existing between elevated reticulated platelet and subsequent CVEs; (2) the quality of included studies; and (3) the sources of heterogeneity, if any.

## Methods

### Search Strategy and Study Selection

We performed a systematic search on Web of Science, PubMed, Scopus, and Embase from inception to January 2020 without language restriction. The major search terms were as follows: reticulated platelets, immature platelet fraction, immature platelet count, platelet turnover, mortality, death, cardiovascular death, coronary artery disease, acute coronary syndrome, myocardial infarction, percutaneous coronary intervention, stroke, and revascularization. Specific search strategy is shown in the Supplementary Materials. Eligible studies met the following criteria:

1. Study types: observational studies (prospective or retrospective studies);
2. Population: adult patients were diagnosed with CAD;
3. Index prognostic factor: RP was the single biomarker that we reviewed for its prognostic value, and the laboratory parameters included either IPF% or IPC;
4. Comparator prognostic factors: the focuses were on the adjusted and unadjusted prognostic value of RPs. No comparator factor was considered when summarizing the unadjusted prognostic effect of RPs, since the unadjusted value of RPs was directly calculated from the absolute events. However, the adjusted value of RPs means the prognostic effect of RPs after adjusting for other conventional prognostic factors (17). Adjustment of the following conventional prognostic factors was predefined as *a priori* of interest to facilitate judgment: age, sex, smoking status, diabetes, hypertension, and dyslipidemia.
5. Outcomes: the clinical outcome of interest included cardiovascular death, non-fatal myocardial infarction (MI), non-fatal cerebrovascular accidents (CVA) (18), and unplanned revascularization (RVA).
6. Timing: the value was measured after CAD diagnosis.

Data search and study selection were performed by two investigators separately (Zhao Y and Lai R). Inconsistencies regarding the decision of the two reviewers were resolved by consensus. Any remaining disagreement was adjudicated to the senior consultant (Zhang Y). Current systematic review and meta-analysis were performed according to the Preferred Reporting Items for Systemic Reviews and Meta-analyses (PRISMA) and Meta-analyses of Observational Studies in Epidemiology (MOOSE) checklist (19, 20) and has been registered in PROSPERO database (registration number: CRD42020169417).

### Data Extraction

Data from eligible studies were extracted by two investigators independently (Zhao Y and Lai R). The discrepancy between the two reviewers during data extraction was resolved by a third supervisor (Zhang Y). We identified the name of first author, year of publication, number of participants, type of cases, proportion of female, proportion of smoker, proportion of patients with hypertension, diabetes mellitus (DM), and dyslipidemia at baseline, antiplatelet medications at baseline, and follow-up duration.

The outcomes were cardiovascular death, myocardial infarction, cerebrovascular accidents, and unplanned revascularization. Mean or median RPs with associated interquartile in patients with and without outcomes were extracted. To assess the prognostic value of RPs after adjustment of conventional factors, we extracted the type of statistical model, adjusted risk estimates and corresponding 95% confidence interval (95% CI), and number of covariates from the original studies. To calculate the unadjusted risk estimates, the absolute number of CVEs was also extracted. The Engauge Digitizer was used to extract the absolute number of events from the published Kaplan–Meier analysis, if sufficient data for meta-analysis were not reported in the primary studies.

### Quality Assessment

Based on Quality in Prognostic Factor Studies (QUIPS) checklists (21), we evaluated the risk of bias of included studies across six domains: study participation, study attrition, prognostic factor measurement, outcome measurement, adjustment for other prognostic factors, and statistical analysis and reporting. For the study confounding part, the following conventional prognostic factors were predefined as *a priori* to facilitate judgment: age, sex, smoking status, diabetes, hypertension, and dyslipidemia. The overall risk of bias in each study was determined as described previously (22).

### Statistical Analysis

For studies with dichotomous outcomes, the level of RPs was classified as high or low according to cut-point reported by original studies. If studies dichotomized the level RPs into two categories, we used the original categories. If there were more than two categories, we recategorized into high and low groups for ease of pooling. Adjusted risk estimates of CVEs reported directly in the included studies were extracted and transformed to the natural logarithms, as described previously (23). The standard errors (SE) were also calculated using the calculator of Review Manager 5.3. Inverse variance method was used to weigh the natural logarithms of adjusted risk estimates and associated standard errors. Unadjusted results reported as count data were presented for composite, cardiovascular death and non-fatal myocardial infarction, cerebral events, and unplanned revascularization. These count data were extracted from original studies and pooled to calculate risk ratio (RR) and 95% CI. Mantel–Haenszel method was used to weigh the unadjusted data.

Considering the potential heterogeneity of prognostic studies as suggested previously (24), we chose to use a random-effects model. Heterogeneity was assessed by Cochrane's *I*^2^ statistic. Sources of heterogeneity were explored by meta-regression model. Subgroup analysis was performed based on laboratory parameters of RPs, follow-up duration, and the number of adjustment variables. We performed funnel plot, Duval's trim and fill method, and Egger's test to assess small study (including publication) bias (25).

Statistical analysis was performed using Review Manager 5.3 (The Cochrane Collaboration, Oxford, UK) and R 3.6.3 (R Core Team, Vienna, Austria). Two-sided tests with *P-*value < 0.05 were considered statistically significant except for heterogeneity assessment in which *P* < 0.10 were used as a significance set.

## Results

### Study Population and Characteristics

**Figure 1:**
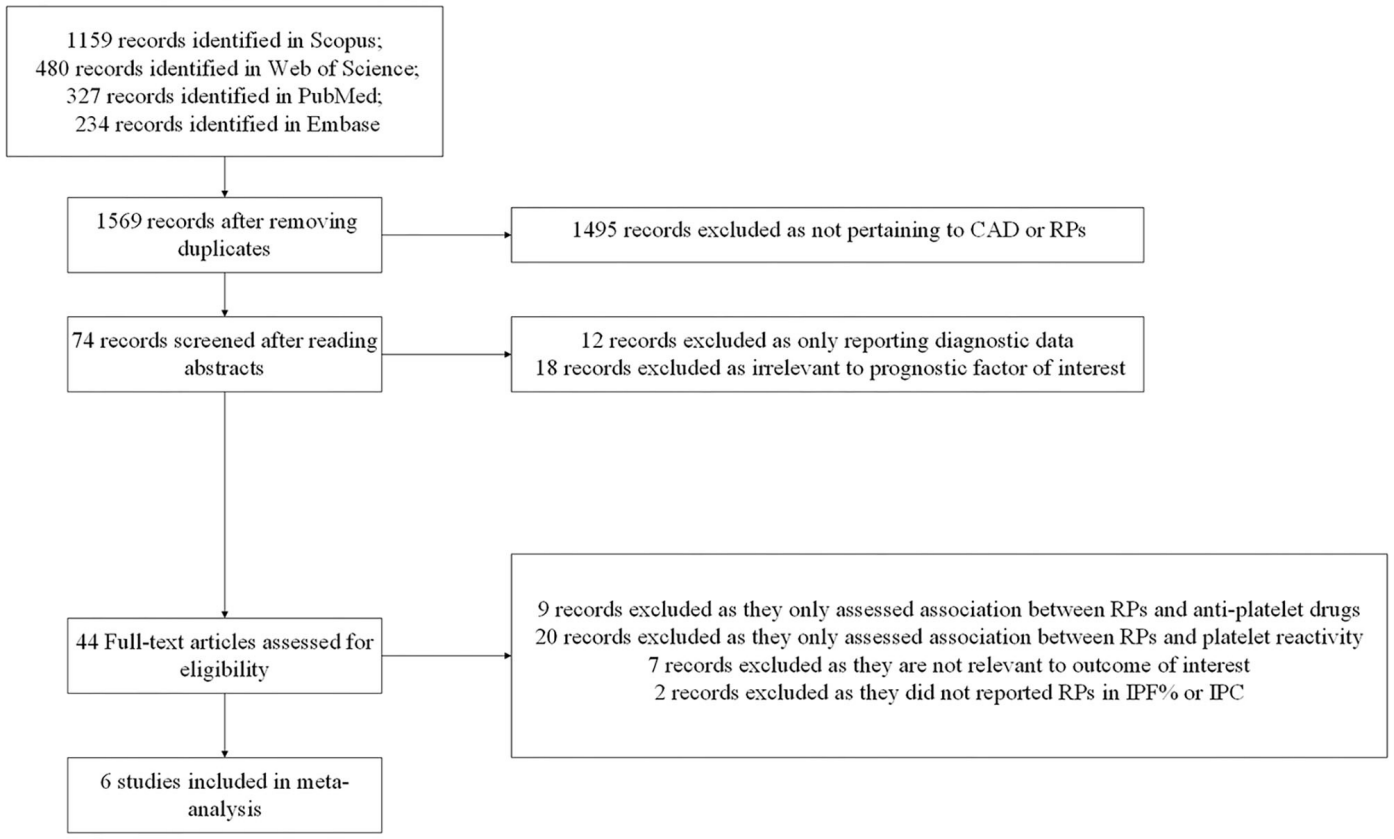
Flow diagram for study selection. CAD, coronary artery disease; RPs, reticulated platelets; IPF%, immature platelet fraction; IPC, immature platelet count.

We identified 1,159 publications from Scopus, 480 publications from Web of Science, 327 publications from PubMed, and 234 publications from Embase. After applying eligibility criteria to 1,569 studies, we included 6 observational studies into the systematic review and meta-analysis. A detailed flowchart of studies inclusion was provided in Figure 1.

Included studies involved a total of 1,636 patients with CAD with 294 outcome events during follow-up. Four of the included studies took place in Europe (26–29), and the remaining two studies were conducted in Iran (30) and the USA (31), respectively. All studies used the prospective cohort study design. The earliest study (31) had a sample size of 51–100 people, and the rest of the studies in the following years had >100 people (26–30). Three studies included patients that underwent post-percutaneous coronary intervention (PCI) (26, 27, 29), one study included stable coronary artery disease (SCAD) combined with diabetes mellitus (DM) as the type of case (30), one study included patients hospitalized with acute coronary syndrome (ACS) (28), and the last one included patients with CAD patients (31). All included studies used the Sysmex XE-2100 automated hematology system to measure RPs, whose results would be expressed as IPF% or IPC. Four studies reported IPF% (27–30), and two studies reported IPC (26, 31). The median of follow-up varied from the in-hospital admission to 5.8 years. The characteristics of the included studies are summarized in Table 1.

**Table 1:** Characteristics of included studies.

**Source**	**Freynhofer et al. (27)**	**Tscharre et al. (26)**	**Cesari et al. (29)**	**López-Jiménez et al. (28)**	**Perl et al. (30)**	**Ibrahim et al. (31)**
Country	Austria	Austria	Italy	Spain	Israel	USA
Type of cases	Post-PCI	Post-PCI	Post-PCI	ACS	SCAD with DM	CAD
Participants (*n*)	486	477	229	251	104	89
Laboratory parameter	IPF%	IPC	IPF%	IPF%	IPF%	IPC
Methodologies of RPs	Sysmex XE-2100	Sysmex XE-2100	Sysmex XE-2100	Sysmex XE-2100	Sysmex XE-2100	Sysmex XE-2100
Measuring time	6–24 h after PCI	6–24 h after PCI	24–48 h after PCI	In the morning of the first day of hospitalization	N/A	Within 72 h of admission to the hospital
Median of follow-up	190 days	5.8 years	1 years	In-hospital admission	2 years	31 months
Total CVEs (*n*)	86	110	22	31	15	30
Reported outcomes of interest	Death, MI, RVA, CVA	Death, MI, CVA	Death	Death	MI, RVA, CVA	Death, MI, RVA
RPs in CVEs	4% [2.9–5.4]	N/A	3.7% [2.4–5.0]	6.60% [4.20–10.80]	4.57%	5.3% [4.3–6.4]
RPs in non-CVEs	3.3% [2.4–4.7]	N/A	2.8% [1.9–4.1]	4.80% [3.10–6.95]	2.53%	3.7% [3.0–5.1]
*P-*value	0.013	N/A	0.05	0.002	<0.001	0.007

*RPs, reticulated platelets; CAD, coronary artery disease; CVE, cardiovascular events; ACS, acute coronary syndrome; PCI, percutaneous coronary intervention; DM, diabetes mellitus; 95% CI, 95% confidence interval; N/A, not applicable; IPF%, immature platelet fraction; IPC, immature platelet count; MI, myocardial infarction; CVA, cerebrovascular accidents; RVA, revascularization*.

Five out of six studies reported that patients with CVEs had a significantly higher RPs level than patients without CVEs. As shown in Table 1, due to the skewed distribution of RPs in the original studies, four studies descripted the RPs as median and associated interquartile range and used the Mann-Whitney *U* test for comparison between two groups (27–29, 31). Only one study provided the means without mentioning standard deviances and used *t*-test to compare continuous variables (30). Therefore, we did not pool the mean differences to perform the meta-analysis. Besides CVEs, two studies reported conflicting results concerning the association between RPs and the risk of bleeding events. Perl reported after adjustment of covariables, the odds ratio (OR) was 0.292 (95% CI, 0.111–0.767), while Freynhofer reported the adjusted OR was 1.211 (CI 95%, 1.042–1.406).

Age of patients ranges from 64 to 76 years. The proportion of female, smoker, and patients with hypertension, diabetes mellitus, and dyslipidemia are 30.6, 36.6, 75.3, 34.3, and 68.8%, respectively. Four studies only enrolled patients taking dual antiplatelet medications (26, 27, 29, 31). One study only enrolled patients treated with a single antiplatelet agent—aspirin or clopidogrel (30). In the last study, 97% of the enrolled patients took aspirin and 75% took clodiprogrel (28). Detailed baseline characteristics of the study populations are provided in Table 2.

**Table 2:** Baseline characteristics of study participants.

**Source**	**CAD [*n* (%)]**	**Mean age**	**Female**	**Smoking**	**Hypertension**	**Diabetes mellitus**	**Dyslipidemia**	**Antiplatelet therapy**
			**[*n* (%)]**	**[*n* (%)]**	**[*n* (%)]**	**[*n* (%)]**	**[*n* (%)]**	**(%)**
Tscharre et al. (26)	477 (100.0)	64.3	149 (31.2)	293 (61.4)	410 (86)	139 (29.1)	376 (78.8)	Dual: aspirin and clodipogrel (100)
Perl et al. (30)	104 (100.0)	71.2	24 (23.1)	13 (12.6)	87 (83.7)	104 (100.0)	94 (90.4)	Single: aspirin or clodipogrel (100)
López-Jiménez et al. (28)	251 (100.0)	68	66 (26.3)	63 (25)	104 (41)	82 (33)	110 (44)	Aspirin (97) or clodipogrel (75)
Cesari et al. (29)	229 (100.0)	76	75 (32.8)	69 (30.1)	131 (57.2)	57 (24.9)	88 (38.4)	Dual: aspirin and clodipogrel (100)
Ibrahim et al. (31)	89 (100.0)	68.1	32 (40.0)	22 (24.7)	84 (94.4)	37 (41.6)	76 (85.4)	Dual: aspirin and clodipogrel (100)
Freynhofer et al. (27)	486 (100.0)	64	154 (31.7)	140 (28.8)	416 (85.6)	140 (28.8)	381 (78.4)	Dual: aspirin and clodipogrel (100)

### Quality Assessment

Two studies indicated moderate quality (28, 31), and the remaining four studies were rated as high quality (26, 27, 29, 30). For the study confounding part, all included studies have adjusted at least one of predefined *a priori* into the multivariable analysis. Two studies have involved all six conventional prognostic factors into adjustment (26, 27). The number of confounding variables varies from 5 to 41. Detailed quality assessments were provided in Supplementary Table 1.

We found that asymmetry existed in the funnel plot. The trim and fill method and Egger's test identified publication bias (Supplementary Figures 1–3). However, in addition to publication bias, the trim and fill method does not take into account other causes for funnel plot asymmetry, and it also performs poorly when substantial heterogeneity exists (32). Various alternative reasons for funnel plot asymmetry should be considered. For example, heterogeneity might cause small-study effects. Therefore, as described previously (24, 33), it is not easy to entangle small study bias from heterogeneity in a single review. Interestingly, as shown in Table 3, we found smaller studies performed the multivariable analysis using fewer adjustment factors, then larger prognostic factor effects might be reported in such studies, rather than caused by publication bias.

**Table 3:** Statistical models of included studies.

**Source**	**Model**	**Cut-off**	**Adjusted risk estimates**	**Unadjusted risk estimates**	**No. of covariates**	**Other platelet function tests**	**Other independent predictors from multivariable analysis**
Tscharre et al. (26)	U&M Cox ph	IPC > 7,600/μL	HR, 1.693 (95% CI, 1.156, 2.481)	HR, 1.716 (95% CI, 1.152, 2.559)	31	MEA, VASP-P, MPV	Age, hyperlipidemia, peripheral artery disease, ACEI or ARB, DES
Ibrahim et al. (31)	M Cox ph	IPC > 7,632/μL	HR, 4.65 (95% CI, 1.78, 12.16)	N/A	7	MPV, LTA	N/A
Perl et al. (30)	M regression	IPF% > median	OR, 1.968 (95% CI, 1.1128–3.432)	N/A	15	MPV	Age, prior MI, anemia
López-Jiménez et al. (28)	M regression	IPF% > 6.2%	OR, 2.42 (95% CI, 1.08, 5.43)	N/A	5	N/A	Admittance Killip
Cesari et al. (29)	U&M regression	IPF% > 3.3%	OR, 2.83 (95% CI, 1.14–7.06)	OR, 4.15 (95% CI, 1.24–13.91)	8	MPV, LTA, H-IPF	H-IPF
Freynhofer et al. (27)	U&M regression	IPF% > 3.35%	OR, 1.136 (95% CI, 1.001–1.288)	OR, 1.173 (95% CI, 1.040–1.324)	41	MEA, VASP-P, MPV	Troponin I, CRP, prior MI

*M regression, multivariable logistic regression analysis; U&M regression, univariable and multivariable logistic regression analysis; U&M Cox ph, univariable and multivariable Cox proportional hazard analysis; M Cox ph, multivariable Cox proportional hazard analysis; HR, hazard ratio; OR, odds ratio; CI, confidence interval; MPV, mean platelet volume; H-IPF, highly fluorescent immature platelet fraction; MEA, multiple electrode aggregometry; LTA, light transmission aggregometry; VASP-P, vasodilator-stimulated phosphoprotein-phosphorylation; ACEI, angiotensin-converting-enzyme inhibitors; ARB, angiotensin II receptor blockers; DES, drug eluting stent; CRP, C-reactive protein*.

### Meta-Analysis

#### Unadjusted Risk Estimates

**Figure 2:**
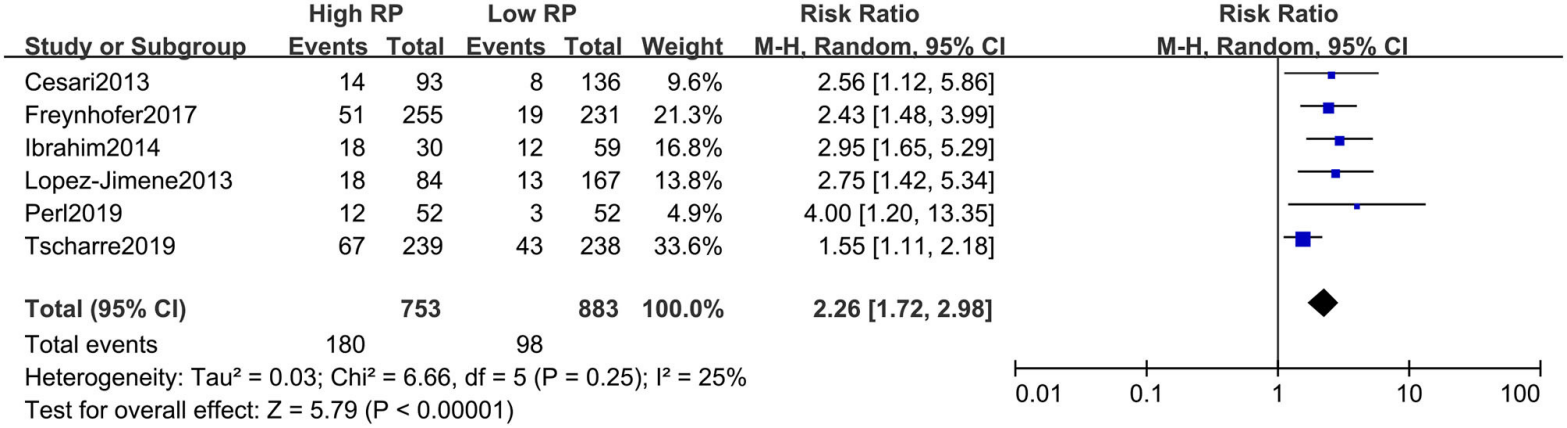
Forest plot for relative risk of composite CVEs in patients with high or low RP level.

**Figure 3:**
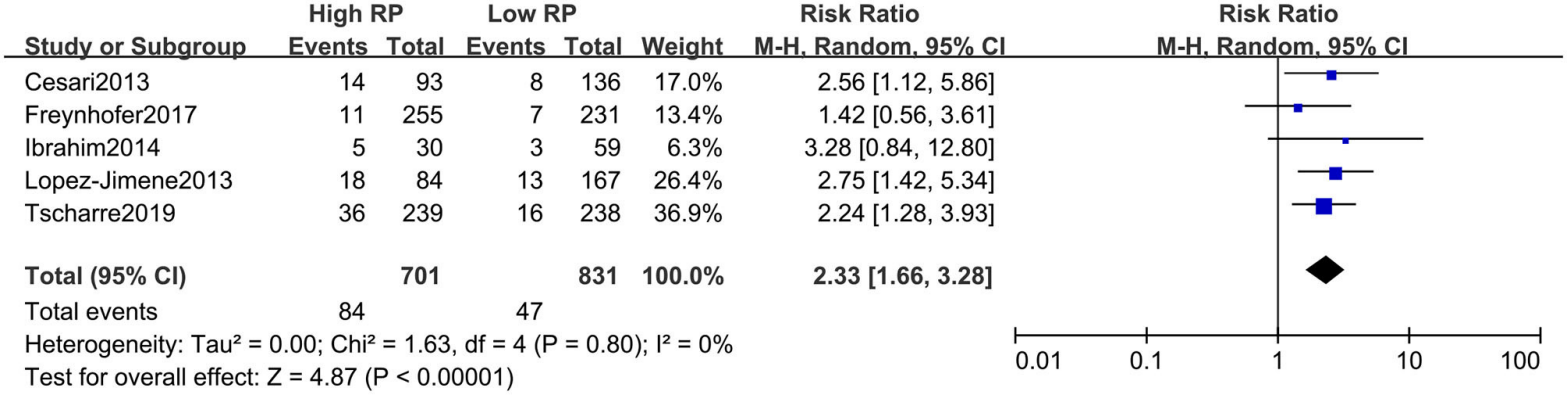
Forest plot for relative risk of cardiovascular death in patients with high or low RP level.

The absolute number of composite events (including fatal and non-fatal) with high and low RPs could be extracted from all included studies (i.e., from a two-by-two table format). For unadjusted risk of CVE, the pooled result of RR for composite CVEs was 2.26 (95% CI, 1.72–2.98) (Figure 2), with little heterogeneity (*I*^2^ = 25%). The summarized RR for fatal CVE was 2.33 (95% CI, 1.66–3.28) (Figure 3), which suggested elevated RPs level is associated with a 133% higher risk of fatal CVEs with no heterogeneity (*I*^2^ = 0).

**Figure 4:**
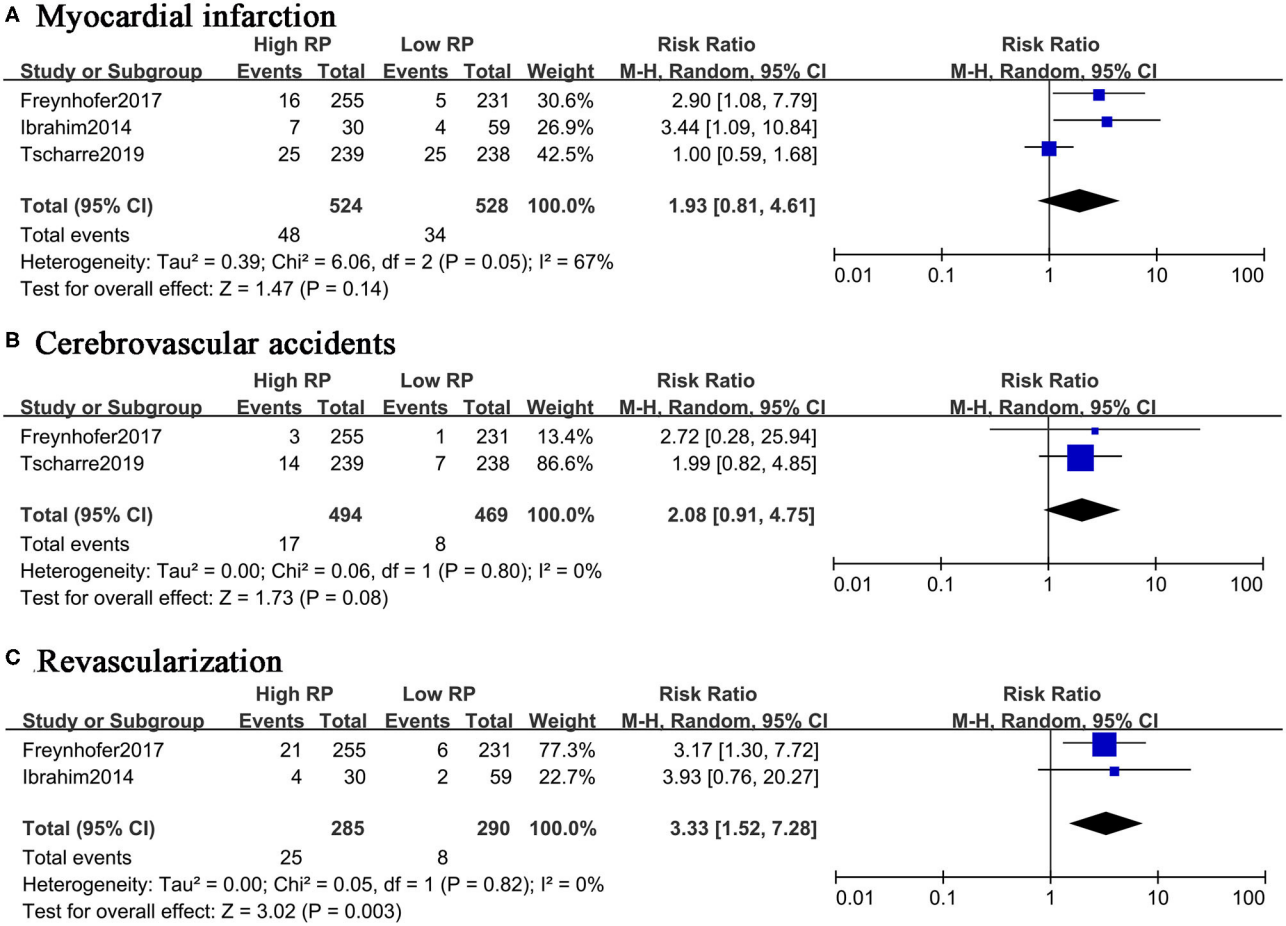
Forest plot for relative risk of non-fatal CVEs in patients with high or low RP level. **(A)** Myocardial infarction. **(B)** Cerebrovascular accidents. **(C)** Revascularization.

Although four studies reported the non-fatal events as the outcomes, the absolute numbers were only available in three studies with the two-by-two table format. The separate meta-analysis suggested that RPs might not act as a prognostic predictor for myocardial infarction (RR, 1.93; 95% CI, 0.81–4.61) or cerebrovascular events (RR, 2.08; 95% CI, 0.91–4.75) (Figures 4A,B). Elevated RPs seem to predict unplanned RVA in the future (RR, 3.33; 95% CI, 1.52–7.28) (Figure 4C). In summary, the elevated level of RPs is associated with high risk of composite events and cardiovascular death, but RPs might not be a good predictor for MI or CVA.

#### Statistical Models and Adjusted Risk Estimates

Two studies used the Cox proportional hazard analysis and reported adjusted hazard ratios (HR) (26, 31). The remaining studies used the logistic regression (27–30) and reported adjusted OR. Four studies measured IPF% and the optimal cut-point varied from 3.3 to 6.2% (27–30). Two studies reported that the cut-point of IPC was around 7.6 × 103/μL (26, 31). Although the number of adjustment covariates varied considerably among included studies, all of them adjusted for at least one of the predefined conventional prognostic factors.

Five out of six studies (26, 27, 29–31) also measured other platelet function tests, namely MPV, another possible prognostic predictor of CVEs (15, 16). However, none of them reported that MPV could act as an independent predictor of CVEs. Platelet activity tests as measured by light transmission aggregometry (LTA) or multiple electrode aggregometry (MEA) did not predict CVEs, either. Similarly, in two studies measuring vasodilator-stimulated phosphoprotein phosphorylation (VASP-P) at baseline, VASP-P did not act as an independent predictor. These findings suggest that RPs are better chronic predictors of events than MPV, LTA, MEA, or VASP-P in patients with CAD.

**Figure 5:**
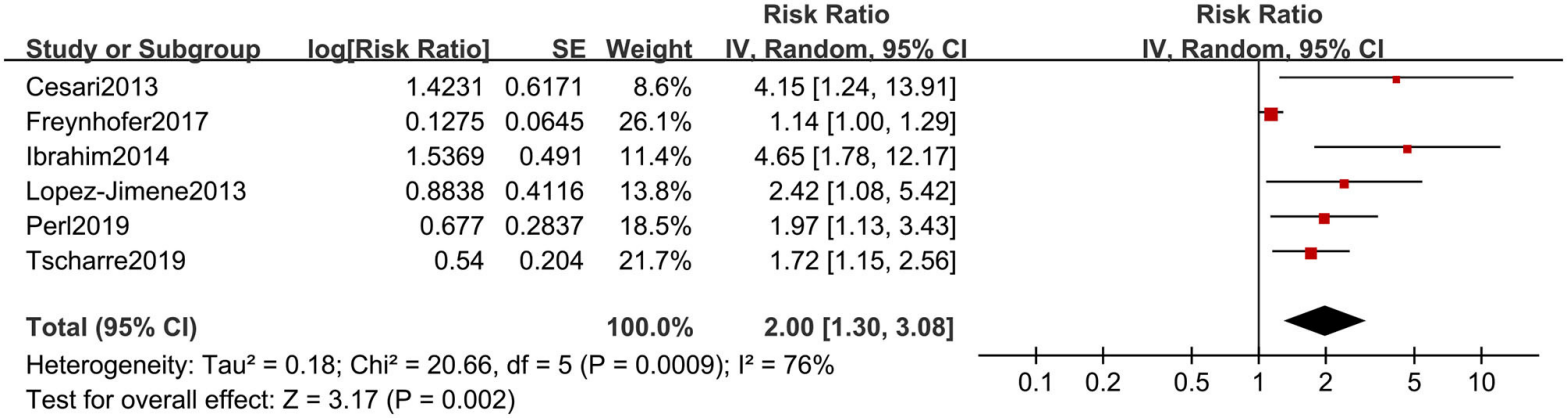
Forest plot for adjusted risk estimates of composite CVEs in patients with high or low RP level.

After transforming the adjusted risk estimates, the summarized result of adjusted risk estimates was 2.00 (95% CI, 1.30, 3.08) (Figure 5). Although the substantial heterogeneity was found, all reported risk estimates from primary studies were in the same direction, which suggested high levels of RPs were associated with CVEs in the future, even after adjustment for conventional prognostic factors.

#### Meta-Regression and Subgroup Analysis

**Figure 6:**
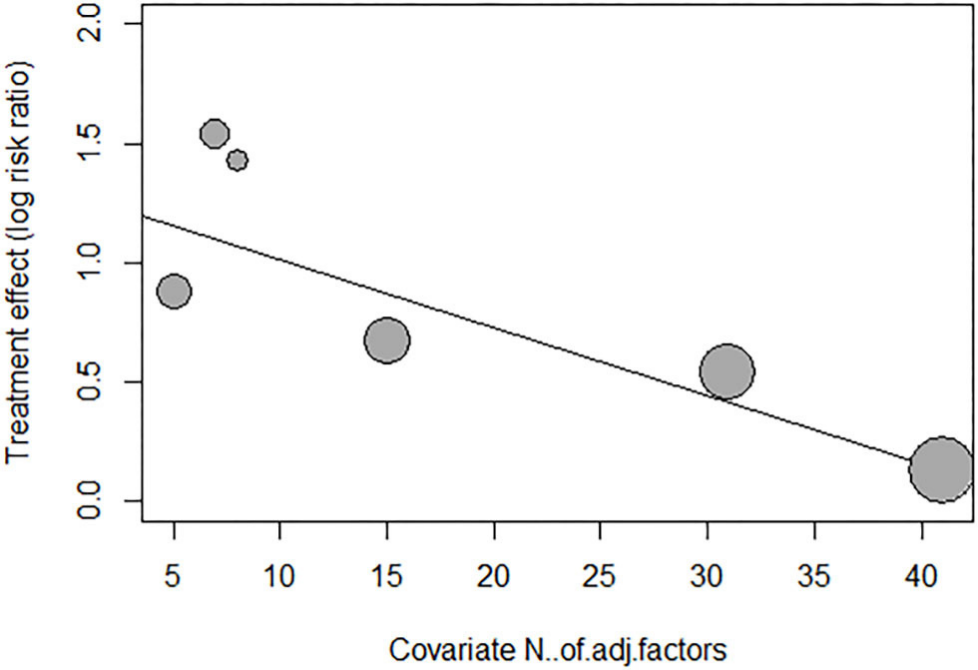
Meta-regression.

We performed meta-regression and subgroup analysis to explore the source of heterogeneity of adjusted risk estimates across studies. As shown in Supplementary Table 2, meta-regression was performed by fitting variables including age, sex, underlying diseases (34), smoking status, duration of follow-up, antiplatelet medications, types of risk estimates, qualities of included studies, and number of adjustment covariates. As shown in Figure 6 and Supplementary Table 2, the meta-regression of adjusted risk estimates suggested that only the number of covariable factors might be the source of heterogeneity (*P* = 0.0052). Moreover, according to the mixed-effect model results, *I*^2^, the estimated amount of residual heterogeneity was reduced to 0 after adjusting for its impact, which means that heterogeneity among studies can be largely attributed to the number of adjustment covariates in different studies. However, the small number of included studies in this review limited us from assuredly concluding the source of heterogeneity.

Like the results of meta-regression, the test for subgroup differences in “number of adjusted covariates” subgroup revealed there is a statistically significant subgroup effect (*P* = 0.01), meaning that the number of adjusted covariates significantly modified the prognostic effect of RPs, although the small number of studies included in this analysis restrained us from confidently concluding there is a true subgroup effect. This analysis also revealed that the significant association between elevated level of RPs and a higher risk of CVE could still be found in “IPF%” and “longer (≥2 years) follow-up” subgroups, which suggested the prognostic effect of RPs were more significant in IPF% and longer (≥2 years) follow-up subgroups than “IPC” and “shorter (<2 years) follow-up” subgroups, respectively. Detailed results of subgroup analyses are provided in Table 4.

**Table 4:** Subgroup analyses on composite cardiovascular events.

**Subgroup**	**Design**	**No. of studies**	**Sample size**	**Test for subgroup difference(*I*^**2**^, *P-*value)**	**Heterogeneity (*I*^**2**^, *P*-value)**	**Meta-analysis (RR, 95% CI)**
Laboratory parameters of RPs	IPF%	4	1,070	0%, *P* = 0.56	72%, *P* = 0.01	1.84 (1.07–3.16)
	IPC	2	566		72%, *P* = 0.06	2.56 (0.98–6.66)
Follow-up	≥2 years	3	670	0%, *P* = 0.81	44%, *P* = 0.17	2.14 (1.36–3.35)
	<2 years	3	966		73%, *P* = 0.02	1.92 (0.88–4.17)
No. of adjusted covariates	≥10	3	1,067	83.3%, *P* = 0.01	71%, *P* = 0.03	1.47 (1.01–2.12)
	<10	3	569		0%, *P* = 0.55	3.35 (1.93–5.81)

*RPs, reticulated platelets; IPF%, immature platelet fraction; IPC, immature platelet count; 95% CI, 95% confidence interval*.

## Discussion

We examined the association between circulating RP levels and cardiovascular outcomes in patients with CAD. The major findings were as follows: (1) The circulating levels of RPs were significantly higher in patients with CVEs, as reported by original studies. (2) In the analysis of adjusted risk estimates, the summarized results demonstrated that increased levels of RPs were associated with a higher risk of CVEs. Moreover, included studies also measured other platelet function tests (including LTA, MEA, and MPV), but none could independently predict CVEs, like RPs. Meta-regression and subgroup analysis demonstrated the different number of adjustment factors in the original studies was the source of heterogeneity, and the prognostic effect of RPs was more significant within groups having follow-up longer than 2 years and expressing laboratory results as IPF%. Publication bias were identified using funnel plot, Egger's test, and trim-and-fill method. (3) The meta-analysis of unadjusted risk estimates also confirmed the prognostic value of RPs for predicting the composite CVEs, cardiovascular death, and RVA. However, RPs might not be a good predictor for MI or CVA.

Besides other conventional risk factors like age and sex, platelets also play a pivotal role in cardiovascular diseases. High on-treatment reactivity might arise due to platelet turnover and lead to poor prognosis of patients with CAD. Our work found that RPs, the novel parameter of platelet turnover, might have the potential as an independent predictor for CVEs. A broad array of platelet turnover tests, namely MPV is already available in clinical practice. Previous meta-analysis and subgroup analysis reported MPV could act as a prognostic marker in patients with ACS other than patients that underwent post-PCI or SCAD (16). Since various conventional cardiovascular risk factors might influence the value of MPV (35) and the afore-mentioned meta-analysis only reported unadjusted risk estimates, it is difficult to clarify the actual prognostic value of MPV. In contrast, our present work reported adjusted and unadjusted risk estimates. Both of them suggested the association between RPs and CVEs. None of the included studies reported that MPV could act as another independent predictor of CVEs after adjusting covariables. Furthermore, MPV only reflects the average size of the whole platelet population and indirectly implies the state of platelet turnover, but not all large platelets are newly formed platelets with enhanced thrombotic activity. In contrast, as a direct parameter reflecting the rate of platelet turnover, RPs represent platelets with larger sizes, increased RNA content, more dense granules, and enhanced pro-hemostatic capacity than mature platelets (36–39). Therefore, it is more likely to help monitor platelet turnover if the measurement of RPs is incorporated into standard blood tests, compared with that of MPV.

The addition of RPs into current prediction models may improve the risk stratification for adverse cardiovascular events in patients with CAD. Currently, there are several prediction models available to enhance prognostication in the management of patients with CAD. For example, the Global Registry of Acute Coronary Event (GRACE) is recommended to predict mortality for patients with ACS by applying risk factors, cardiac biomarkers, and electrocardiograms. As summarized extensively, GRACE score's prognostic value is not perfect, and incremental effects after adding other laboratory parameters have been reported (40, 41). For example, platelets play a pivotal role in the pathogenesis of all types of CAD. Improved predictive value of GRACE score after combination with platelet reactivity or MPV has been reported (42, 43). Although one study found RPs had the potential as an independent predictor even among patients who were not considered high risk using GRACE score (28) and one study involved the GRACE score into adjustment of multivariable analysis (29), none of them assessed the combined predictive value of RPs and GRACE score. Large-scale observational studies are required to assess the combined predictive value.

Monitoring of RP levels may also help assess the response to antiplatelet therapies. Included studies reported relatively uniform antiplatelet medications, aspirin and clopidogrel. Aspirin constitutes the critical component of secondary prevention of CAD and is complemented by clopidogrel in patients undergoing PCI (44). One of the plausible mechanisms that RPs could act as a predictor of CVEs is that newly formed RPs undermine antiplatelet therapies, namely those with pharmacokinetically short lives, like aspirin and clopidogrel (8, 10, 45). Both of them irreversibly bind their targets (COX-1 and P2Y_12_) but have short half-lives. The variability in response to antiplatelet medications might be associated with different platelet turnover rates, which also means different renewal rates of drug targets (COX-1 and P2Y_12_). In contrast, the blockade by the reversible inhibitor of P2Y_12_, ticagrelor, is closely related to the circulating drug concentration (38). Bernlochner et al. demonstrated that as a surrogate parameter of platelet turnover, RPs show a greater impact on platelet reactivity in response to prasugrel, an irreversible inhibitor of P2Y_12_, compared with ticagrelor, a reversible inhibitor of P2Y_12_ (46). Armstrong also reported that RPs play a role in hyporesponse to clopidogrel but not ticagrelor (10). In summary, for aspirin and clopidogrel, other than reversible P2Y_12_ inhibitors, the diminished impacts on platelet activation could be explained by the minimal exposure to active metabolites of newly released RPs. The application of antiplatelet therapies comes with an increased risk of bleeding (47). Two studies also reported the association between RP levels and bleeding events. However, they found conflicting results (27, 30). Large prospective studies are necessary to answer this important research question.

Given the advent of a fully automated cell analyzer, the values of RPs become simple to obtain and easy to interpret from automatic cell counters (5). Currently, in some developed countries, RP determination is a standardized methodology and easy to perform like MPV. For included studies evaluating long-term results, the reported cut-off was similar for IPC (around 7,600/μL) and IPF% (around 3.4%) separately.

Overall, included studies indicated moderate or high quality, and all of them were performed in developed countries. Study objectives and populations were clearly specified, and the valid and reliable measurement of RPs was also reported. All studies determined RPs before cardiovascular outcomes. All studies reported adjusted risk estimates based on appropriate statistical models. However, some limitations must be considered. First, the heterogeneity test of adjusted prognostic values suggested substantial heterogeneity across studies, which might be explained by the different adjustment of prognostic covariates. In addition, the sample size in primary studies are relatively small since RP measurement by the automated analyzer has only been employed for a short time in clinical practice. The identified publication bias also limited the robustness of the results. Therefore, the results should be interpreted with great caution. Based on standardized measurement of RPs, especially IPF%, large-scale observational studies with long follow-up duration and reliable confounding adjustments are needed for drawing a firm conclusion on the prognostic value of RPs in predicting adverse CVEs.

## Conclusions

Our work suggested circulating level of RPs of patients with CAD could be a prognostic factor for CVEs in the future, even after adjustment of conventional covariates. Further large-scale studies with long follow-up duration are still necessary for drawing a firm conclusion of the prognostic value of RPs in patients with CAD.

## Data Availability Statement

The raw data supporting the conclusions of this article will be made available by the authors, without undue reservation.

## Author Contributions

YZhang and DS: conceptualization and supervision. YZhao and RL: data curation, formal analysis, investigation, and methodology. YZhao and YZhang: project administration. YZhao: software and writing (original draft). RL, YZhang, and DS: writing (review and editing). All authors contributed to the article and approved the submitted version.

## Conflict of Interest

The authors declare that the research was conducted in the absence of any commercial or financial relationships that could be construed as a potential conflict of interest.
