# Supplementary material for: The Prognostic Value of Reticulated Platelets in Patients With Coronary Artery Disease: A Systematic Review and Meta-Analysis
Source: Front Cardiovasc Med. 2020 Oct 23;7:578041. doi: 10.3389/fcvm.2020.578041 (PMC7644708; doi:10.3389/fcvm.2020.578041)
Supplement: Supplementary file 1 [file Presentation_1.PPTX]

## Slide 1
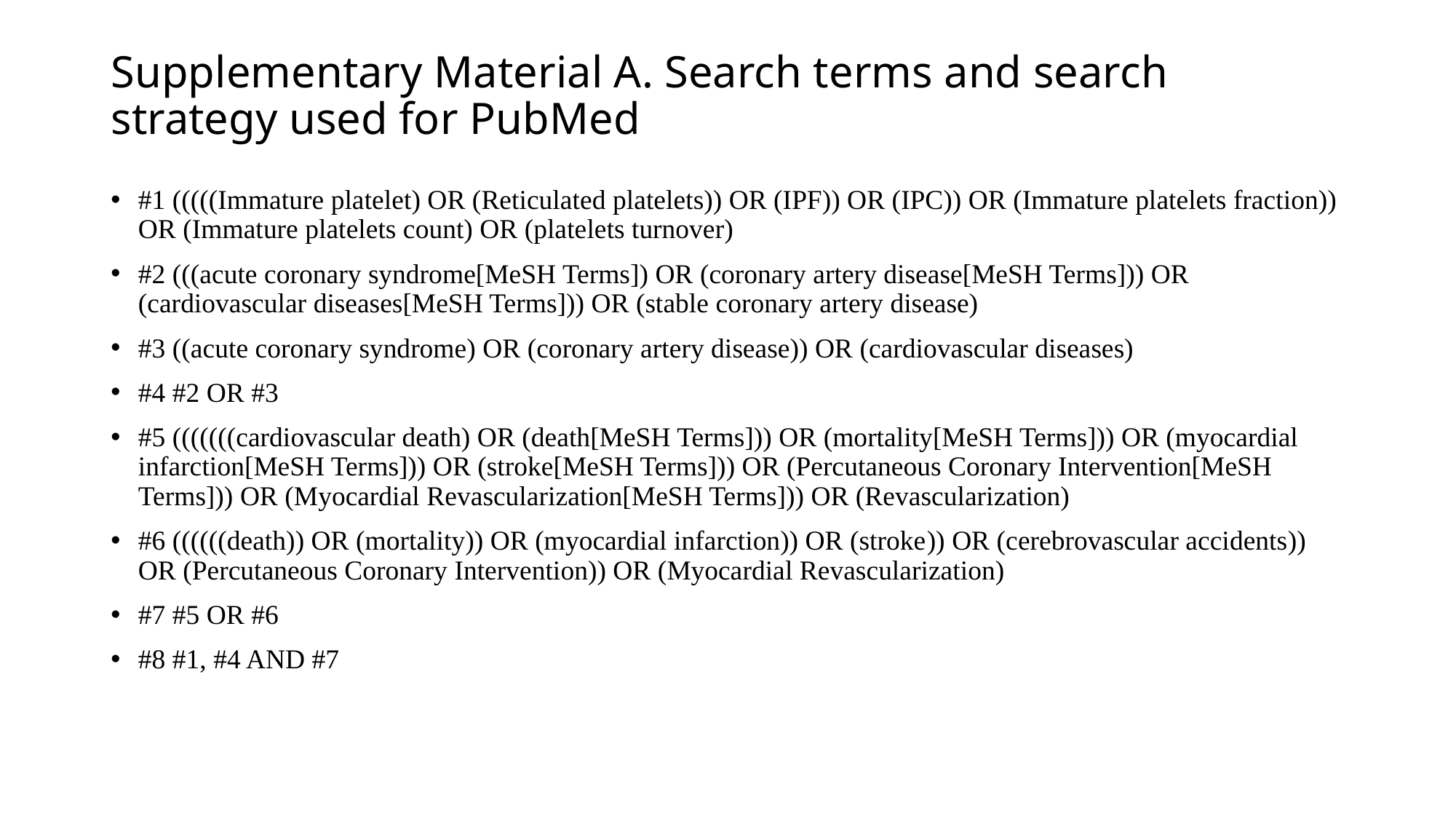

# Supplementary Material A. Search terms and search strategy used for PubMed
#1 (((((Immature platelet) OR (Reticulated platelets)) OR (IPF)) OR (IPC)) OR (Immature platelets fraction)) OR (Immature platelets count) OR (platelets turnover)
#2 (((acute coronary syndrome[MeSH Terms]) OR (coronary artery disease[MeSH Terms])) OR (cardiovascular diseases[MeSH Terms])) OR (stable coronary artery disease)
#3 ((acute coronary syndrome) OR (coronary artery disease)) OR (cardiovascular diseases)
#4 #2 OR #3
#5 (((((((cardiovascular death) OR (death[MeSH Terms])) OR (mortality[MeSH Terms])) OR (myocardial infarction[MeSH Terms])) OR (stroke[MeSH Terms])) OR (Percutaneous Coronary Intervention[MeSH Terms])) OR (Myocardial Revascularization[MeSH Terms])) OR (Revascularization)
#6 ((((((death)) OR (mortality)) OR (myocardial infarction)) OR (stroke)) OR (cerebrovascular accidents)) OR (Percutaneous Coronary Intervention)) OR (Myocardial Revascularization)
#7 #5 OR #6
#8 #1, #4 AND #7

## Slide 2
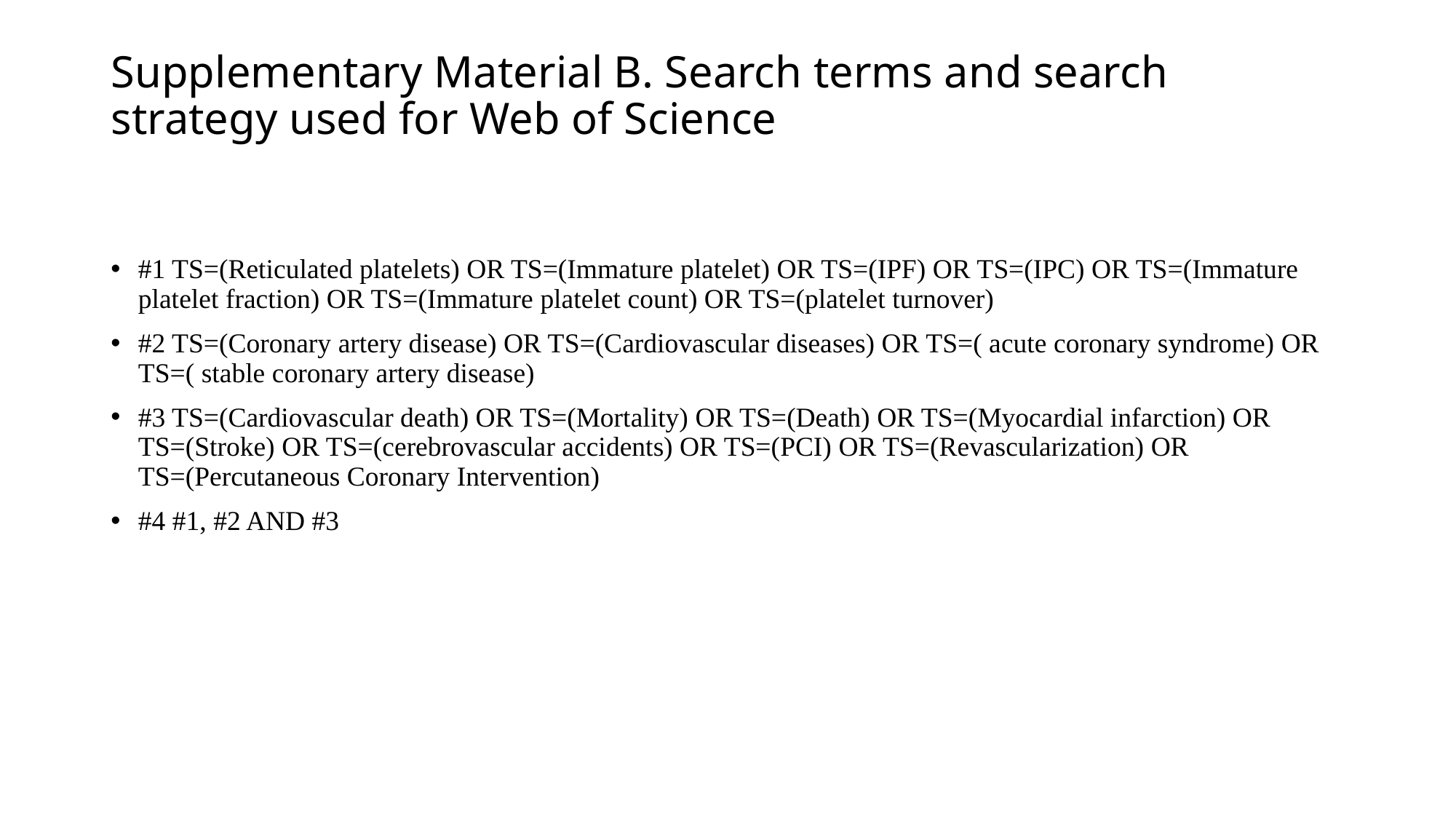

# Supplementary Material B. Search terms and search strategy used for Web of Science
#1 TS=(Reticulated platelets) OR TS=(Immature platelet) OR TS=(IPF) OR TS=(IPC) OR TS=(Immature platelet fraction) OR TS=(Immature platelet count) OR TS=(platelet turnover)
#2 TS=(Coronary artery disease) OR TS=(Cardiovascular diseases) OR TS=( acute coronary syndrome) OR TS=( stable coronary artery disease)
#3 TS=(Cardiovascular death) OR TS=(Mortality) OR TS=(Death) OR TS=(Myocardial infarction) OR TS=(Stroke) OR TS=(cerebrovascular accidents) OR TS=(PCI) OR TS=(Revascularization) OR TS=(Percutaneous Coronary Intervention)
#4 #1, #2 AND #3

## Slide 3
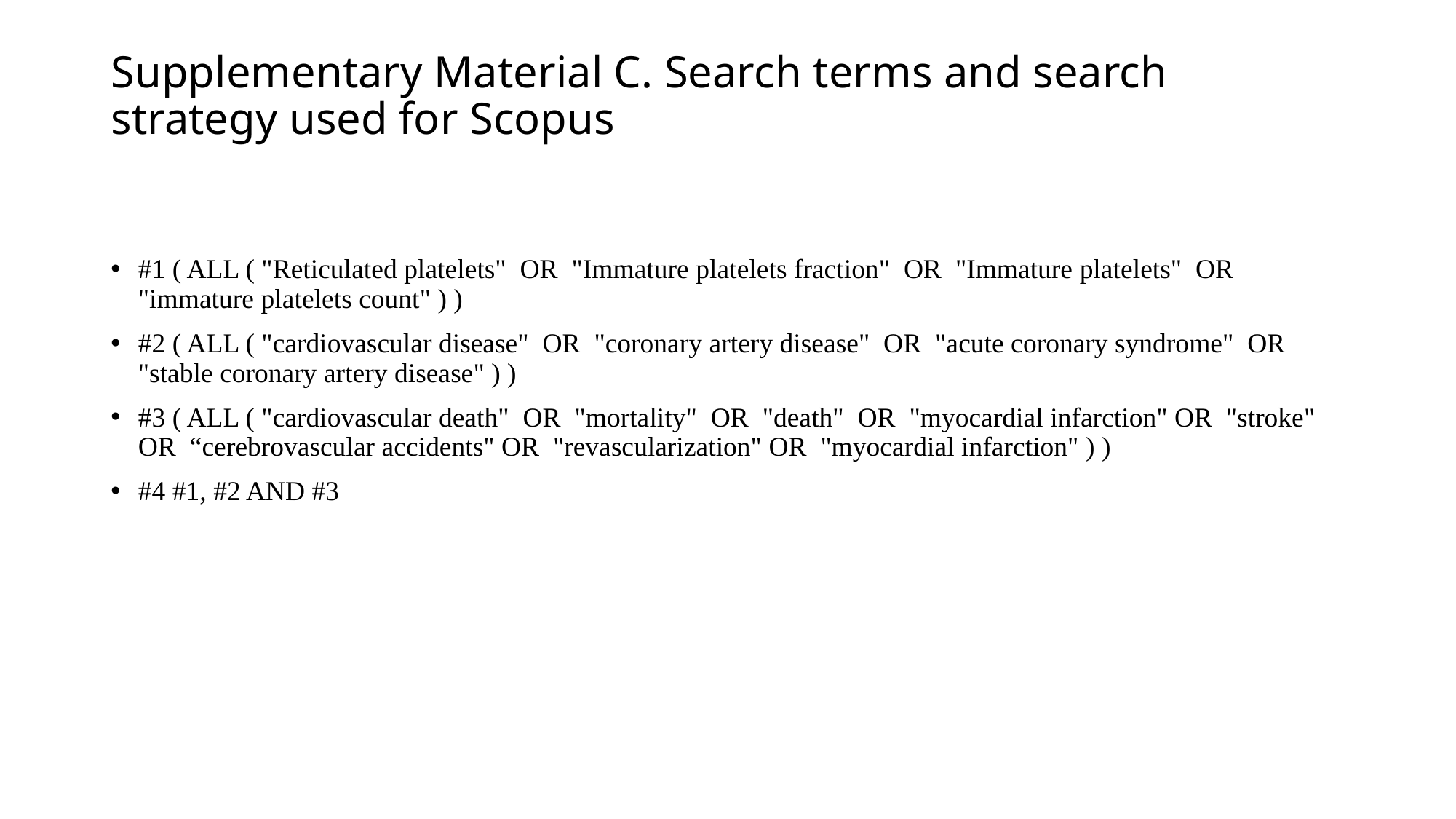

# Supplementary Material C. Search terms and search strategy used for Scopus
#1 ( ALL ( "Reticulated platelets" OR "Immature platelets fraction" OR "Immature platelets" OR "immature platelets count" ) )
#2 ( ALL ( "cardiovascular disease" OR "coronary artery disease" OR "acute coronary syndrome" OR "stable coronary artery disease" ) )
#3 ( ALL ( "cardiovascular death" OR "mortality" OR "death" OR "myocardial infarction" OR "stroke" OR “cerebrovascular accidents" OR "revascularization" OR "myocardial infarction" ) )
#4 #1, #2 AND #3

## Slide 4
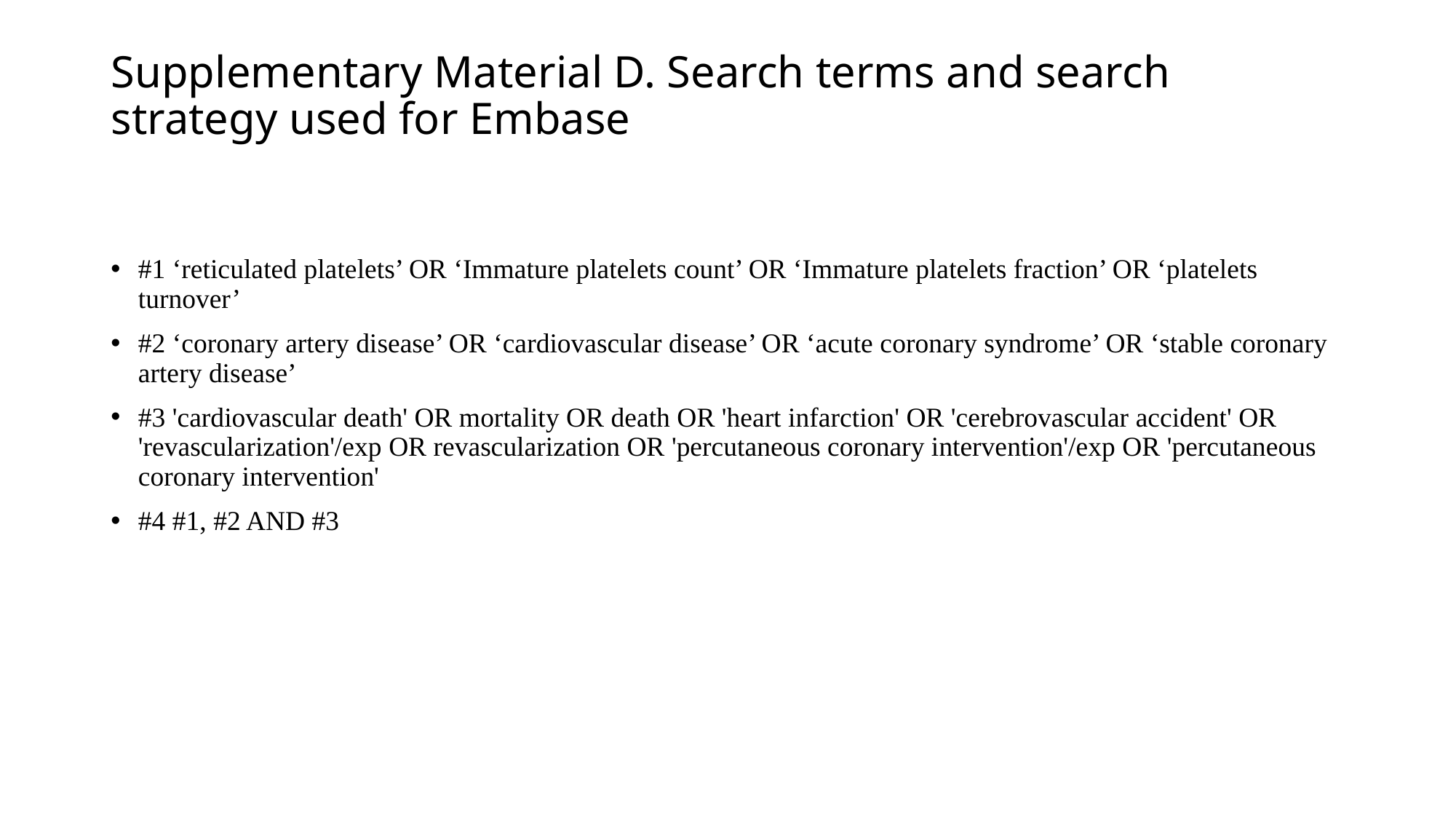

# Supplementary Material D. Search terms and search strategy used for Embase
#1 ‘reticulated platelets’ OR ‘Immature platelets count’ OR ‘Immature platelets fraction’ OR ‘platelets turnover’
#2 ‘coronary artery disease’ OR ‘cardiovascular disease’ OR ‘acute coronary syndrome’ OR ‘stable coronary artery disease’
#3 'cardiovascular death' OR mortality OR death OR 'heart infarction' OR 'cerebrovascular accident' OR 'revascularization'/exp OR revascularization OR 'percutaneous coronary intervention'/exp OR 'percutaneous coronary intervention'
#4 #1, #2 AND #3

## Slide 5
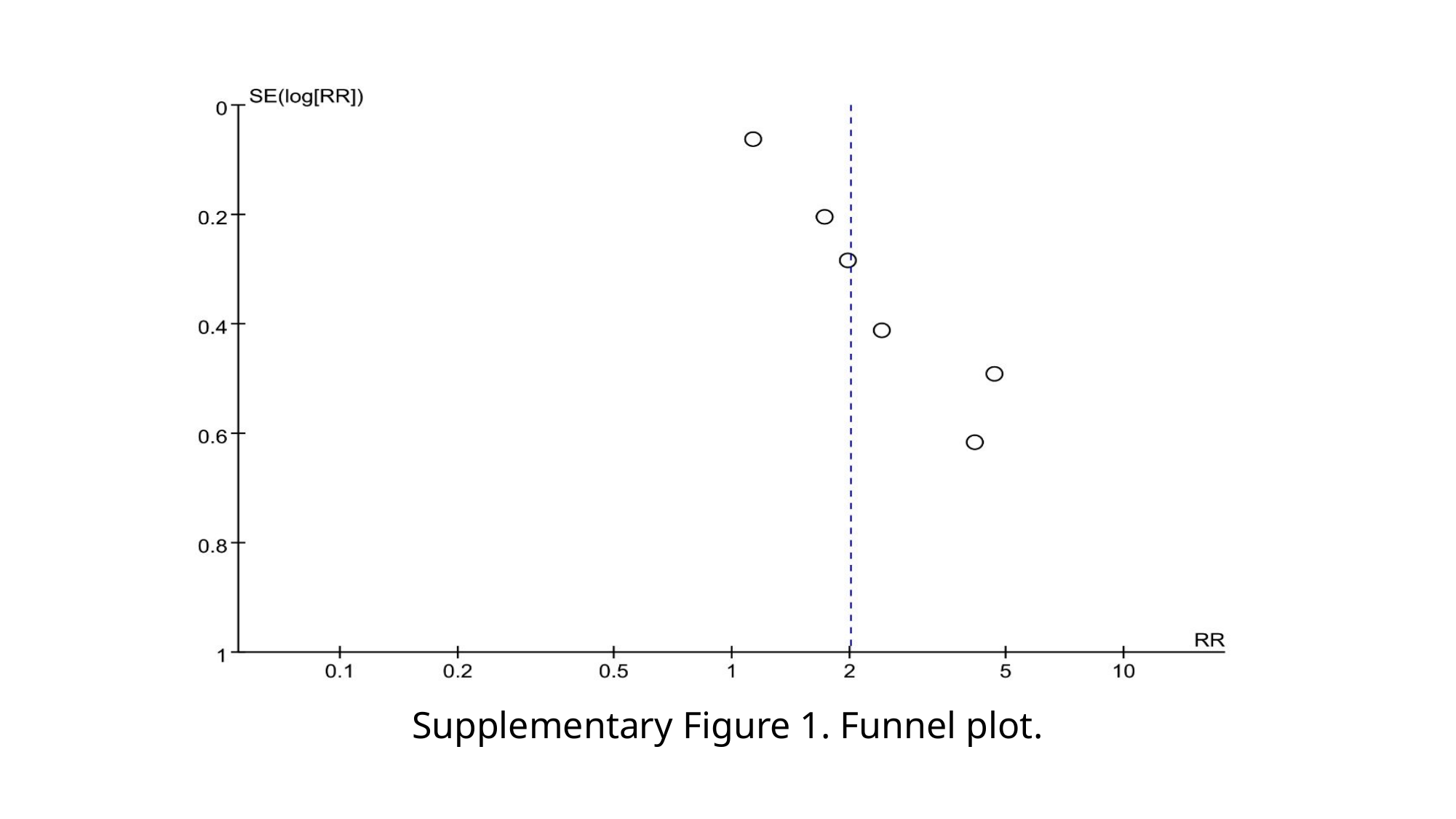

Supplementary Figure 1. Funnel plot.

## Slide 6
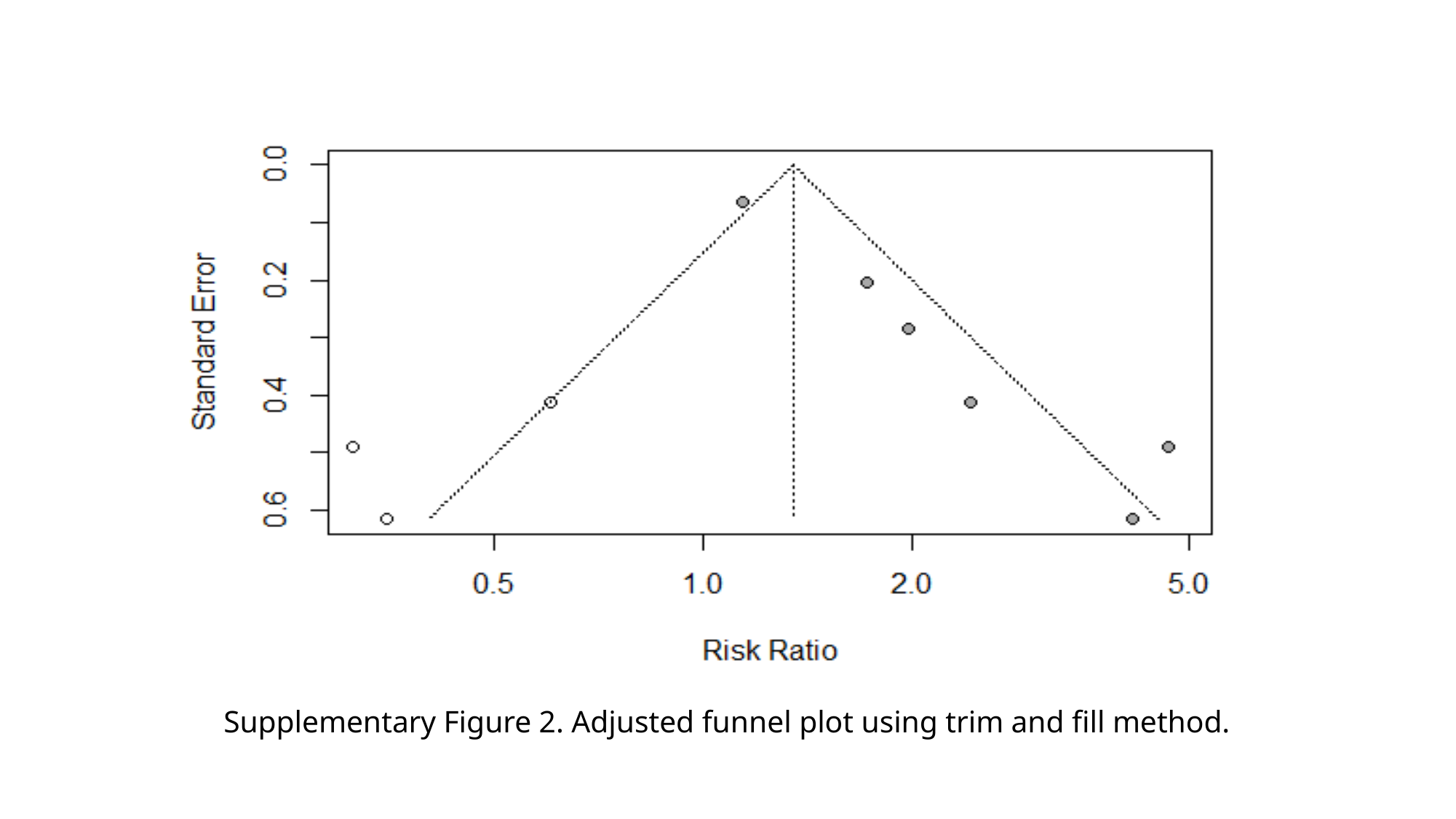

Supplementary Figure 2. Adjusted funnel plot using trim and fill method.

## Slide 7
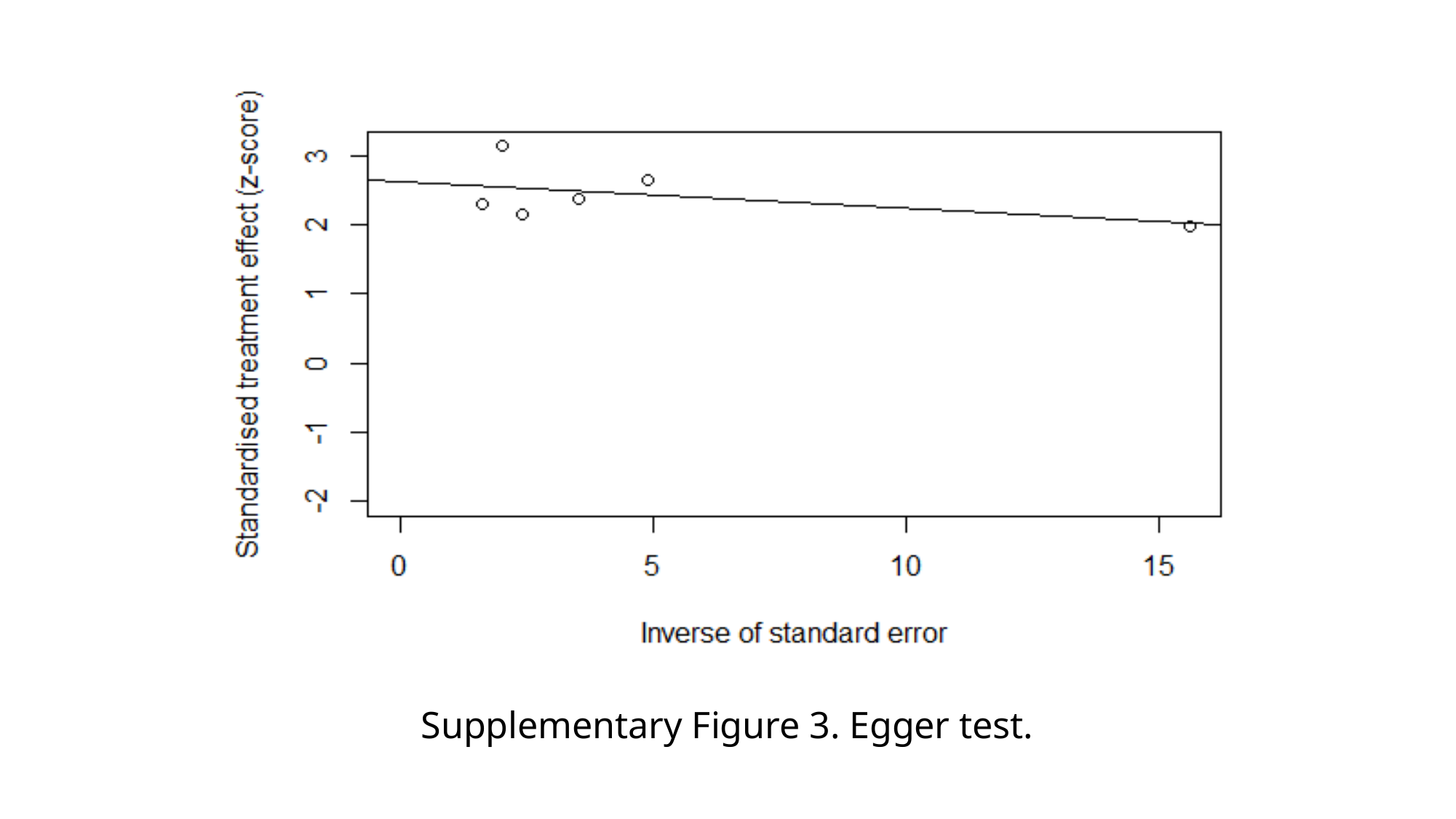

Supplementary Figure 3. Egger test.

## Slide 8
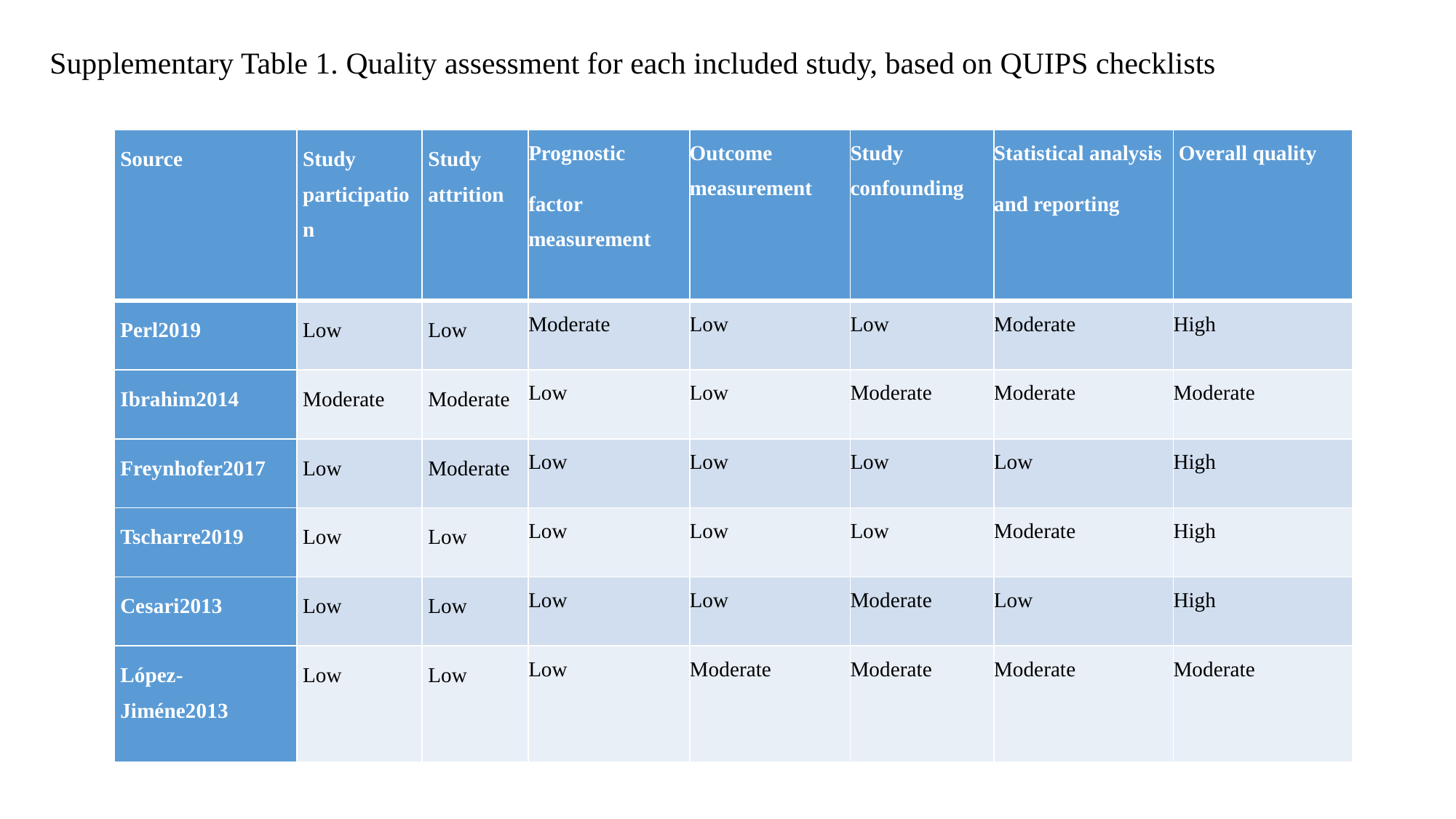

# Supplementary Table 1. Quality assessment for each included study, based on QUIPS checklists
| Source | Study participation | Study attrition | Prognostic factor measurement | Outcome measurement | Study confounding | Statistical analysis and reporting | Overall quality |
| --- | --- | --- | --- | --- | --- | --- | --- |
| Perl2019 | Low | Low | Moderate | Low | Low | Moderate | High |
| Ibrahim2014 | Moderate | Moderate | Low | Low | Moderate | Moderate | Moderate |
| Freynhofer2017 | Low | Moderate | Low | Low | Low | Low | High |
| Tscharre2019 | Low | Low | Low | Low | Low | Moderate | High |
| Cesari2013 | Low | Low | Low | Low | Moderate | Low | High |
| López-Jiméne2013 | Low | Low | Low | Moderate | Moderate | Moderate | Moderate |

## Slide 9
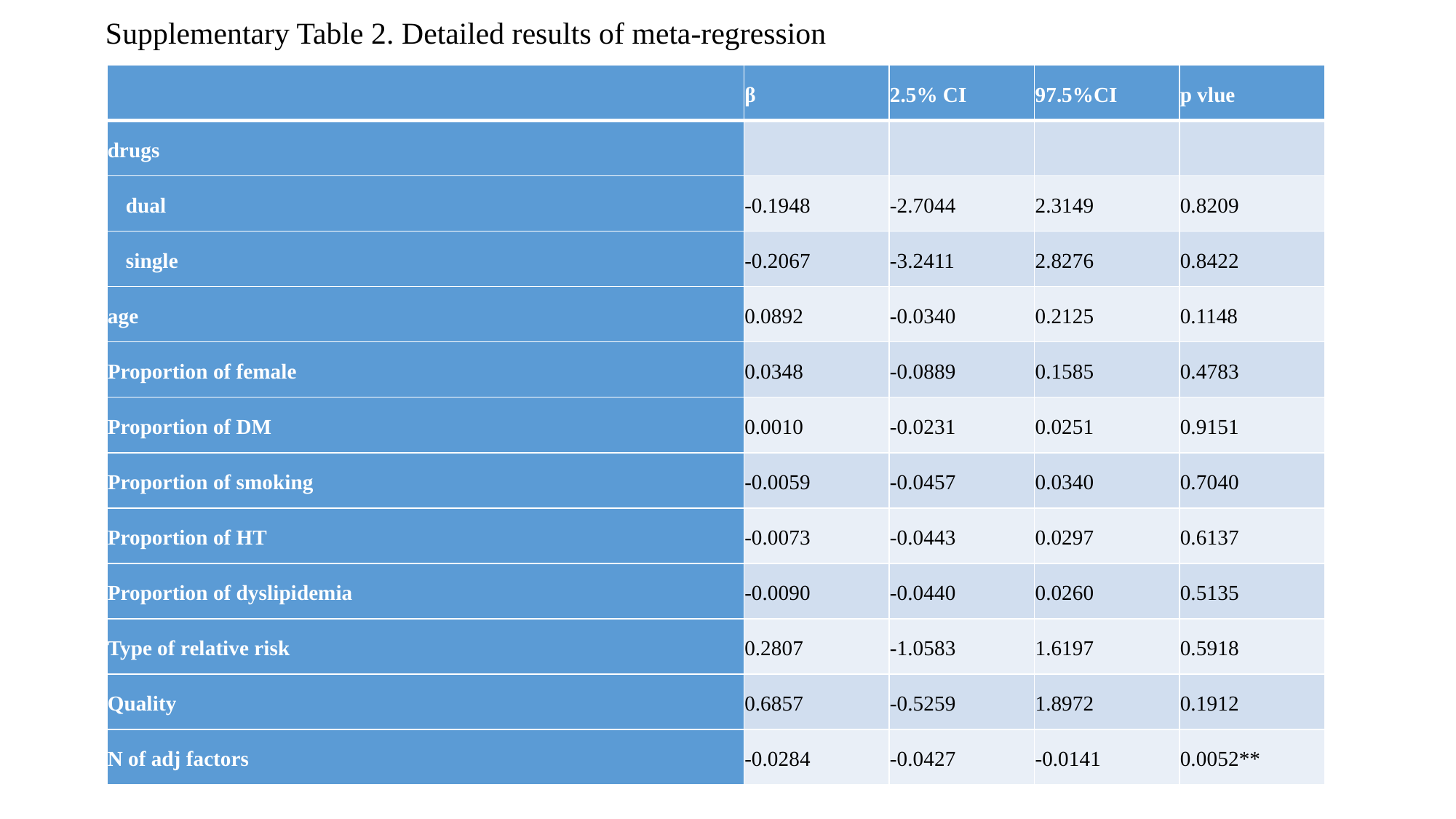

# Supplementary Table 2. Detailed results of meta-regression
| | β | 2.5% CI | 97.5%CI | p vlue |
| --- | --- | --- | --- | --- |
| drugs | | | | |
| dual | -0.1948 | -2.7044 | 2.3149 | 0.8209 |
| single | -0.2067 | -3.2411 | 2.8276 | 0.8422 |
| age | 0.0892 | -0.0340 | 0.2125 | 0.1148 |
| Proportion of female | 0.0348 | -0.0889 | 0.1585 | 0.4783 |
| Proportion of DM | 0.0010 | -0.0231 | 0.0251 | 0.9151 |
| Proportion of smoking | -0.0059 | -0.0457 | 0.0340 | 0.7040 |
| Proportion of HT | -0.0073 | -0.0443 | 0.0297 | 0.6137 |
| Proportion of dyslipidemia | -0.0090 | -0.0440 | 0.0260 | 0.5135 |
| Type of relative risk | 0.2807 | -1.0583 | 1.6197 | 0.5918 |
| Quality | 0.6857 | -0.5259 | 1.8972 | 0.1912 |
| N of adj factors | -0.0284 | -0.0427 | -0.0141 | 0.0052\*\* |
